# Electronic Modulation of Cu Catalytic Interfaces by Functionalized Ionic Liquids for Enhanced CO_2_ Reduction

**DOI:** 10.3390/molecules30112352

**Published:** 2025-05-28

**Authors:** Chuanhui Wang, Wei Zhou, Jiamin Ma, Zhi Wang, Congyun Zhang

**Affiliations:** 1 School of Environment and Geography, Qingdao University, Qingdao 266071, China; wch2022020768@163.com (C.W.); 13770594749@163.com (W.Z.); majiamin07@163.com (J.M.); 2 School of Materials Science and Engineering, North University of China, Taiyuan 030051, China

**Keywords:** CO_2_RR, ionic liquids, DFT, interface charge reconstruction, C–C coupling

## Abstract

The electrocatalytic CO_2_ reduction reaction (CO_2_RR) into value-added multi-carbon C_2+_ products holds significant promise for sustainable chemical synthesis and carbon-neutral energy cycles. Among the various strategies developed to enhance CO_2_RR, the use of ionic liquids (ILs) has emerged as a powerful approach for modulating the local microenvironment and electronic structure of Cu-based metal catalysts. In this study, to unravel the molecular-level mechanisms underlying these enhancements, density functional theory calculations (DFTs) were employed to systematically explore how ILs with different terminal groups modulate the electronic reconstruction of the Cu surface, further affecting the *CO–*CO coupling and product selectivity. Electronic structure analyses reveal that ILs bearing polar moieties (–SH, –COOH) can synergistically enhance the interfacial electron accumulation and induce an upshift of the Cu d-band center, thereby strengthening *CO adsorption. In contrast, nonpolar IL (CH_3_) exhibits negligible effects, underscoring the pivotal role of ILs’ polarity in catalyst surface-state engineering. The free energy diagrams and transition state analyses reveal that ILs with polar groups significantly lower both the reaction-free energy and activation barrier associated with the *CO–*CO coupling step. This energetic favorability selectively inhibits the C_1_ product pathways and hydrogen evolution reaction (HER), further improving the selectivity of C_2_ products. These theoretical insights not only unveil the mechanistic origins of IL-induced performance enhancement but also offer predictive guidance for the rational design of advanced IL–catalyst systems for efficient CO_2_ electroreduction.

## 1. Introduction

With the growing global demand for sustainable energy and the increasing emphasis on environmental protection [1,2], the electrocatalytic CO_2_ reduction reaction (CO_2_RR) has become a research hotspot [3,4]. Converting CO_2_ into high value-added multi-carbon (C_2+_) products can not only effectively alleviate the greenhouse effect but also achieve the recycling of carbon resources, providing a new approach for the energy transition [5,6]. Copper (Cu)-based materials are among the few metal catalysts capable of driving the electrochemical reduction in CO_2_ toward C_2+_ products such as ethylene and ethanol [7,8]. Its distinct catalytic performance originates from a balanced *CO adsorption energy, which is sufficient to stabilize key reaction intermediates while still allowing for their subsequent C–C coupling. However, Cu-based materials still face several intrinsic challenges in the reduction in CO_2_ to produce C_2_ products. For example, the product selectivity remains unsatisfactory, often yielding a complex mixture of hydrocarbons, which hinders the efficient isolation of a single high-purity target product [9,10]. In addition, there are also problems such as insufficient stability, high overpotential, and severe competition from side reactions such as hydrogen evolution [11,12], all of which collectively compromise the overall efficiency and practicality of Cu-mediated CO_2_ reduction. In order to further enhance its catalytic performance and selectivity towards C_2_ products, researchers have explored various strategies, including surface structure regulation (crystal plane regulation, vacancy engineering, changes in shape, size, roughness, etc.) [13,14,15,16,17], elemental doping (heteroatom doping and alloying) [18,19,20,21], regulation of the local microenvironment [22,23], as well as organic molecule modification (polymer binders or functional additives) [24,25]. Among these, the ionic liquid (IL)-modified system has shown great potential due to its unique customizable physical and chemical properties, including high electrochemical stability, excellent ionic conductivity, and good CO_2_ solubility. In addition, the adjustable hydrophobicity of ILs can enhance the affinity for CO_2_ and suppress the hydrogen evolution reaction (HER) [26,27].

ILs can achieve the microstructural regulation of Cu-based catalysts. For instance, 1-octyl-3-methylimidazolium chloride ([Omim]Cl) can act as a bifunctional reagent to prepare cuprous oxide nanoparticles with rough surfaces and oxygen vacancies [28]. These nanoparticles with such a unique structure are highly conducive to the adsorption of intermediates and facilitate the formation of C_2+_ products. In addition, ILs can also be used as electrolytes to regulate the interfacial microenvironment in electrochemical systems. ILs form a structured electric double layer at the electrode–electrolyte interface through the self-assembly of their cations and anions under applied potential [29,30]. This interfacial structure in turn influences the adsorption, stabilization, and transformation of CO_2_RR intermediates [31,32,33,34]. Particularly, imidazolium-based ILs can form Im–CO_2_ adducts with CO_2_ reduction intermediates, thereby enhancing CO_2_ adsorption and activation. Theoretical calculations corroborate these findings by revealing that ILs reduce the Gibbs free energy barriers associated with key intermediates and strengthen their adsorption energies, ultimately promoting more efficient catalytic activity.

ILs have exhibited remarkable functional advantages by virtue of the optimization of the catalyst structure and the regulation of the interfacial microenvironment. The adjustability of their cationic and anionic components has opened up broad space for the further precise control of catalytic activity and product selectivity. Shaikh and co-workers conducted kinetic calculations and demonstrated that varying amino acid-based anions influence the cycloaddition reaction with CO_2_ and modulate electrostatic interactions [35]. The rational functionalization of Cu-based catalysts has been validated to facilitate for the direct cleavage of the C–O bond, thus maintaining a high selectivity for C_2_H_4_. Comparative studies on Cu electrodes modified with polyacrylic acid (PAA, with –COOH group) and polyvinylidene fluoride (PVDF, with –CF_2_ group) reveal that Cu–PAA favors the formation of formic acid rather than Cu–PVDF (functionalized with –CF_2_ group), whereas the Cu–PVDF system predominantly yields methane [36]. Moreover, adjusting the anionic components of polymerized ionic liquids (PILs) has been demonstrated to effectively modulate product distribution, highlighting the potential of molecular design strategies in steering CO_2_RR selectivity [37]. Although the above examples illustrate that replacing the cations and anions in ionic liquids can accelerate the reaction and improve the reaction selectivity, most of these studies only remain at the macroscopic level of experiments. As for how the replacement of cations and anions affects the adsorption capacity of CO_2_ step by step, as well as the subsequent coupling, bond-breaking, and hydrogenation processes, there is a lack of in-depth research on the specific principal mechanisms in between. We are in urgent need of a means at the molecular level to uncover the essence of these influences, starting from the source of charges.

In the context of IL-modulated CO_2_ electroreduction, density functional theory calculations (DFTs) [38] offer a robust theoretical framework for elucidating reaction pathways, quantifying energy barriers, identifying active sites, and capturing interfacial electronic interactions. For example, DFT calculations have demonstrated that the strong *CO absorption ability on [BPy]BF_4_ is due to the charge accumulation around F^−^ [39]. Moreover, DFT analyses reveal that ILs can promote the *CO dimerization, a critical process in the formation of C_2+_ products [40]. Under certain IL-modified conditions, *CHO–*CHO coupling has also been identified as a thermodynamically favorable C–C bond formation pathway [41]. In addition, DFT can simulate solvent effects, interfacial interactions, and material stability. These insights are essential for understanding how ILs influence intermediate stabilization and transition-state energetics at the catalyst–electrolyte interface, thereby bridging molecular-level interactions with macroscopic catalytic performance and guiding the rational design of IL-integrated electrocatalytic systems.

Although DFT has achieved certain results in establishing the correlation between macroscopic experimental phenomena and reaction mechanisms at the molecular level, in the research field of ILs, there is still a lack of in-depth and comprehensive understanding of how the structural changes in IL functional groups affect the interfacial charge distribution, the stability of intermediates, and the C–C bonding energy barriers. The quantitative relationships between these group substitutions and key catalytic parameters, as well as the regulatory mechanisms of these group substitutions on the electrochemical reaction pathways, have not formed a systematic and complete theoretical framework. Therefore, starting from this point, we will study the influence of the groups on ILs on the catalytic reduction in CO_2_ on the metal surface.

In this context, we focus on exploring the potential mechanisms of the catalytic performance of ILs with different functional groups in promoting the formation of C_2_ products during the CO_2_RR on the surface of Cu. Through the comprehensive application of DFT simulations, we systematically analyzed the microscopic processes of the interactions at the interface between Cu and ILs. The research results show that ILs achieve thermodynamic stability and kinetic acceleration by precisely regulating the interfacial microenvironment. At the electronic structure level, ILs adjust the charge distribution on the surface of Cu. IL (SH) and IL (COOH) transfer electrons to Cu, making the center of the d orbital of Cu closer to the Fermi level. This not only enhances the Cu–C interaction and stabilizes the *CO intermediate but also shortens the distance between C atoms. From an energy perspective, the modification of ILs (excluding IL (CH_3_)) reduces the adsorption energy of intermediates. At the same time, it also reduces the thermodynamic energy difference and kinetic barriers of the C–C coupling step, ensuring the rapid progress of the rate-determining step (RDS) in CO_2_RR. In addition, the interfacial charge microenvironment induced by ILs not only inhibits the conversion of CO_2_ into C_1_ products, thus increasing the selectivity of C_2_ products, but also significantly suppresses the competitiveness of the HER through proton confinement and the inhibition of the *H desorption step. We have determined that Cu–IL (SH) is the most outstanding catalyst, which is due to the fact that the SH group transfers the most charge to Cu. So far, we have established the entire correlation system encompassing the functional groups, charge transfer, intermediate stability, coupling energy barrier, and product selectivity.

## 2. Simulation Details

We selected the Cu (1 0 0) surface [42,43] and designed several ILs with different terminal groups, including the mercapto group, carboxyl group, and methyl group, named 1-(3-mercaptopropyl)-3-methylimidazolium chloride (IL (SH)), 1-(3-carboxylpropyl)-3-methylimidazolium chloride (IL (COOH)), and 1-(3-butyl)-3-methylimidazolium chloride (IL (CH_3_)). These were optimized and then placed on the Cu surface for surface optimization, named as Cu–IL (SH), Cu–IL (COOH), and Cu–IL (CH_3_), respectively. Throughout the process, the Vienna Ab initio Simulation Package. 6.4.2 (VASP. 6.4.2) [44] was used to perform DFT calculations for the CO_2_RR on the Cu surface and the Cu–IL interfaces modified by different types of ILs. The projector-augmented wave (PAW) [45] method was adopted to describe the interactions between ions and electrons, and the generalized gradient approximation (GGA) [46] with the Perdew–Burke–Ernzerhof (PBE) functional was used to calculate the energy and electronic structure properties of the materials. For the Cu (1 0 0) surface, a 6 × 6 supercell was used. To avoid the interactions between periodic units during the structural relaxation process, a vacuum space of more than 20 Å was set. During the optimization process, the bottom two layers of copper atoms were fixed, and other atoms were allowed to relax. To ensure the convergence of the total energy, the kinetic energy cutoff of the plane wave expansion was set to 500 eV, and all calculations used a Monkhorst-Pack 2 × 2 × 1 K-point mesh [47]. The convergence criteria for force and energy were set to 0.01 eV/Å and 10^−5^ eV, respectively. In the frequency calculation, the energy convergence criterion was set to 10^−7^ eV. The electronic analysis used a 4 × 4 × 1 K-point mesh. The Gibbs free energy (ΔG) for each step was calculated using the following formula: ΔG = ΔE + ΔE_ZPE_ − TΔS, where ΔE is the adsorption energy of the adsorbate, T is the temperature (set to 300 K), and ΔE_ZPE_ and ΔS are the differences in zero-point energy and zero-point entropy, respectively. For the transition state calculation, the nudged elastic band (NEB) method was adopted [48], and it was checked whether the transition state had only one imaginary frequency. To obtain the influence mechanism of ILs on the charge distribution of the Cu surface, bader charge and differential charge calculations were carried out to describe the electron distribution on various Cu–IL catalysts and determine the optimal adsorption site of *CO. The adsorption energy of the *CO intermediate on the Cu–IL catalyst was defined as E_ads_ = E_total_ − E_surface_ − E_*CO_, where E _total_ is the energy of the Cu or Cu–IL catalyst adsorbing *CO, E_surface_ is the energy of the Cu surface or the Cu–IL catalyst, and E_*CO_ is the energy of the free *CO. The differential charge density of *CO adsorbed on the catalyst was defined as follows: Δρ = ρ_A/B_ − ρ_A_ − ρ_B_, where ρ_A/B_ is the total charge density of *CO adsorbed on the Cu–IL catalyst, ρ_A_ is the charge density of the model after deleting the *CO intermediate from the total adsorption model, and ρ_B_ is the charge density of the model after removing the Cu or Cu–IL substrate from the total adsorption model.

**Figure 1:**
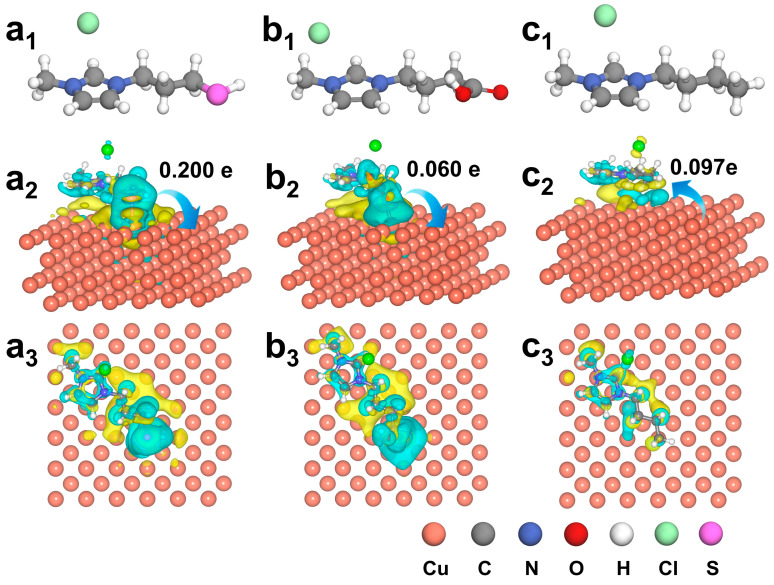
The configuration of (**a_1_**) IL (SH), (**b_1_**) IL (COOH), and (**c_1_**) Cu–IL (CH_3_). The different charges of the ILs and Cu surfaces in three IL-modified catalyst systems, with cyan and yellow areas representing charge depletion and charge accumulation, respectively. (**a_2_**,**a_3_**) Cu–IL (SH), (**b_2_**,**b_3_**) Cu–IL (COOH), (**c_2_**,**c_3_**) the different charges of Cu–IL (CH_3_).

The optimized structures of three ILs with different functional groups are shown in Figure 1a_1_–c_1_. For the adsorption models of the three catalysts on the Cu base, we adhered to the principle of consistency and carried out adsorption at the same site. We designed several models with different bond lengths and bonding forms, optimized them, and found the optimal structures.

## 3. Results and Discussion

### 3.1. Functional Group-Governed Electronic Reconstruction in IL-Modified Cu Catalysts

**Figure 2:**
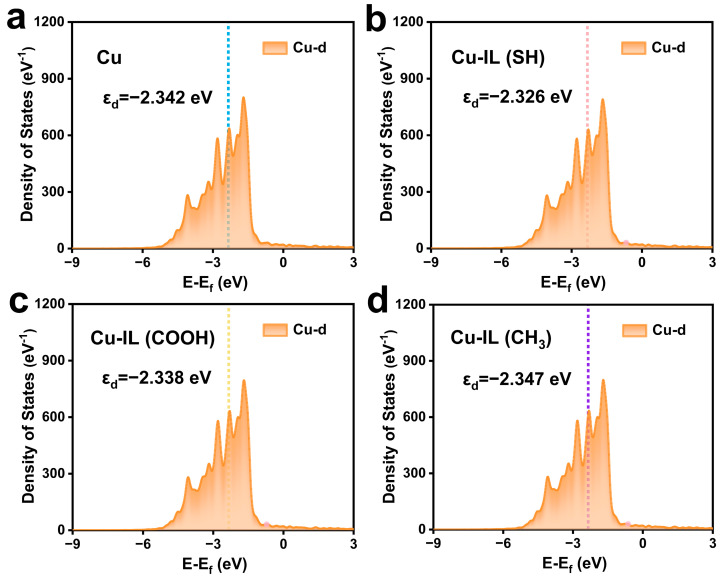
The DOS plots of the d orbital of Cu before *CO adsorption. (**a**) Cu, (**b**) Cu–IL (SH), (**c**) Cu–IL (COOH), and (**d**) Cu–IL (CH_3_).

Based on the top view and side view configurations shown in Figures S1 and S2, the adsorption behavior of ILs on the Cu surface varies significantly with the nature of the functional group. Through systematic bader charge analysis (Figure S3) and differential charge density calculations (Figure 1), we quantitatively established distinct electron transfer patterns between IL modifiers and the Cu substrate. Notably, the Cu–IL (SH) system exhibits the most pronounced charge transfer with a net electron donation of 0.200 e from the IL (SH) to the Cu surface, followed by the Cu–IL (COOH) system, which transferred 0.060 e to the Cu. In contrast, Cu–IL (CH_3_) displays an inverse charge transfer direction with 0.097 e migrating from Cu to the ILs. Bader charge analysis reveals significant interfacial electronic reorganization after the incorporation of different ILs, where Cu–IL (SH) manifests an enhanced electron accumulation surrounding the adsorption sites of Cu, whereas Cu–IL (CH_3_) exhibits distinct electron depletion, demonstrating ligand-specific modulation of surface electron density. These results underscore the critical role of the functional group of the IL cation group in tuning interfacial charge distribution. This interfacial electronic reconstruction was further correlated with d-band center positioning through density of states (DOS) analysis (Figure 2). The calculated d-band center values followed the order of the d-band center size, as follows: Cu–IL (SH) (−2.326 eV) > Cu–IL (COOH) (−2.338 eV) > Cu (−2.342 eV) > Cu–IL (CH_3_) (−2.347 eV). This systematic upshift of the Cu d-band center originates from electron donation by the ILs, which increases the electron occupancy of the Cu 3d orbitals. This charge transfer elevates the energy of antibonding states due to enhanced electron–electron repulsion and modified orbital overlap, thereby upshifting the d-band center. According to the d-band center theory, the elevated d-band center in Cu–IL (SH) suggests stronger adsorbate–substrate interactions due to the increased overlap between catalyst d-orbitals and adsorbate frontier molecular orbitals.

### 3.2. Electronic Structure Modulation and Intermediate Stabilization via IL-Modified Cu Surfaces

The electrocatalytic reduction in carbon dioxide to C_2_ products is governed by two crucial stages: the C–C coupling step and the post-C–C coupling step, which are regarded as the RDS and the selectivity-determining step (SDS), respectively. For the rational design of catalysts, it is essential to understand how these steps are regulated by interfacial interactions. Against this backdrop, we systematically investigated the role of ionic liquids in regulating the adsorption configurations and energies of key intermediates during the electrocatalytic reduction in carbon dioxide. For different systems, we selected the pathway *CO + *CO → *COCO for analysis. We optimized the structures of the initial and final states of the coupling and analyzed data such as the adsorption capacity of the substrate for the intermediates, charge transfer, and transition-state energy barriers. The optimized structural models of the initial and final states of the *CO–*CO coupling are shown in Figures S4–S7.

**Figure 3:**
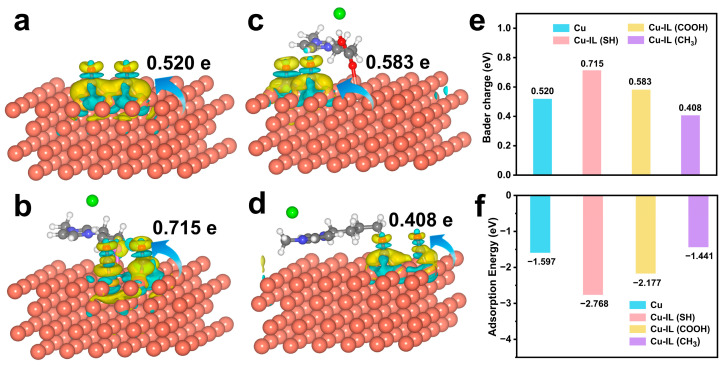
The different charges of the *CO + *CO and Cu/Cu–IL surfaces in pure Cu and three IL-modified catalyst systems in the initial state of C–C coupling. (**a**) The different charges of pristine Cu, (**b**) the different charges of Cu–IL (SH), (**c**) the different charges of Cu–IL (COOH), (**d**) the different charges of Cu–IL (CH_3_). (**e**) The bader charge of *CO + *CO in the initial state of C–C coupling. (**f**) The adsorption energy of *CO + *CO on Cu/Cu–IL surfaces in the initial state of C–C coupling.

We systematically investigated the evolution of the electronic structure of key intermediates on the pristine and IL-modified Cu surfaces. Differential charge density and bader charge analysis (Figure 3a–e and Figure S8) demonstrates ligand-dependent charge redistribution upon *CO adsorption. For the Cu–IL (SH) catalyst, the Cu surface exhibits a charge depletion of approximately 0.715 e to the adsorbed *CO intermediates, compared to 0.520 e on the pristine Cu, indicating an enhanced electron donation capability. This enhancement can be attributed to the electron-donating nature of the –SH group, which facilitates electron density accumulation on the Cu surface and promotes subsequent transfer to the intermediates. In contrast, the –CH_3_ group, being weakly electron-donating and sterically non-polar, leads to reduced electron transfer, with only 0.408 e accumulated on the intermediates—less than that observed for pristine Cu. The –COOH group exhibits a moderate effect, resulting in an intermediate charge accumulation of 0.583 e. These results highlight the critical role of ILs’ functional group’s electronic properties in modulating interfacial charge redistribution and Cu–intermediate coupling strength.

This functional group-dependent charge transfer behavior is closely correlated with the adsorption energy and observed d-band center shifts. The influence of IL functionalization on intermediate stabilization was first evaluated by comparing the adsorption energies of the *CO dimer configuration on different catalysts. The adsorption strength (Figure 3f) follows the following trend: Cu–IL (SH) (−2.768 eV) > Cu–IL (COOH) (−2.177 eV) > Cu (−1.597 eV) > Cu–IL (CH_3_) (−1.441 eV). The significantly more negative adsorption energies for Cu–IL (SH) and Cu–IL (COOH) indicate stronger binding interactions with the intermediates, which enhance *CO surface coverage and reduce the spatial distance between adjacent *CO species, thereby promoting the probability of *CO–*CO coupling. This variation in adsorption energy is intrinsically correlated to the interfacial electronic structure, particularly the extent of charge transfer from the Cu surface to the adsorbates. Electron-donating functional groups such as –SH and –COOH promote substantial electron donation from the Cu substrate to the adsorbates, which facilitates intermediate stabilization. In contrast, the methyl-functionalized ILs suppress both intermediate stabilization and reaction site activation, underscoring their limited ability to activate key intermediates.

**Figure 4:**
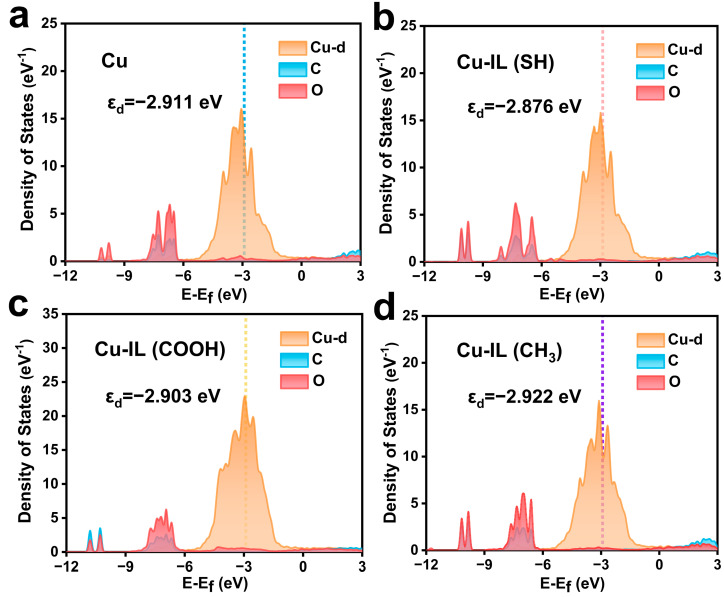
The DOS plots of the d orbitals of Cu and the total orbitals of C and O of adsorbed *CO + *CO in the initial state of C–C coupling. (**a**) Cu, (**b**) Cu–IL (SH), (**c**) Cu–IL (COOH), and (**d**) Cu–IL (CH_3_).

These observed charge transfer characteristics align well with the corresponding shifts in the Cu d-band center (Figure 4). Specifically, Cu–IL (SH) exhibits an upshift of the d-band center from −2.911 eV (pristine Cu) to −2.876 eV, followed by Cu–IL (COOH) (−2.903 eV), whereas Cu–IL (CH_3_) causes a downshift to −2.922 eV, moving the d-band center further away from the Fermi. This trend reflects a weakened orbital overlap and reduced Cu–intermediate interaction. In comparison, the upshifted d-band centers in the Cu–IL (SH) and Cu–IL (COOH) systems bring the Cu d orbitals closer in energy to the molecular orbitals of *CO, thus enhancing orbital hybridization and leading to stronger Cu–C bonding and more favorable adsorption geometries.

**Figure 5:**
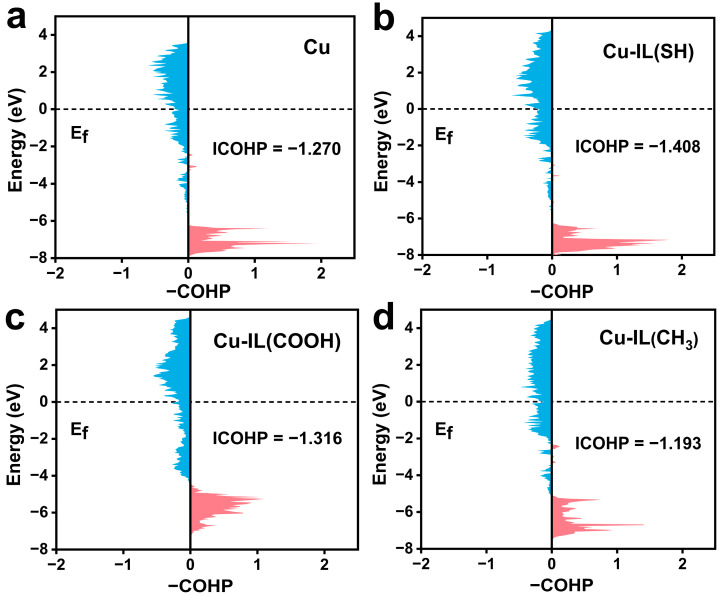
The projected COHP results of the overlap states between the *C atom of *CO + *CO and the adsorbed Cu atoms in the initial state of C–C coupling. (**a**) Cu, (**b**) Cu–IL (SH), (**c**) Cu–IL (COOH), and (**d**) Cu–IL (CH_3_).

To provide further orbital-level insight into Cu–intermediate interactions, we carried out the calculation of the crystal orbital Hamiltonian population (ICOHP) [49] for the Cu–C bonds at the initial stage of *CO–*CO coupling (Figure 5). The ICOHP value of Cu–C in the pure Cu is −1.270 eV, indicating a moderate bonding strength. Upon IL modification, the Cu–C ICOHP becomes more negative for Cu–IL (SH) (−1.408 eV) and Cu–IL (COOH) (−1.316 eV), signifying stronger bonding interactions. Conversely, Cu–IL (CH_3_) exhibits a less negative value of −1.193 eV, further confirming weakened orbital overlap and diminished Cu–C coupling.

These electronic structure trends are closely correlated with the corresponding geometric configurations. At the initial state of C–C coupling (Table 1), the Cu–C bond lengths shorten from 1.999 Å on pristine Cu to 1.975 Å and 1.990 Å in the Cu–IL (SH) and Cu–IL (COOH) systems, respectively, while extending to 2.016 Å in Cu–IL (CH_3_). A similar trend is observed for the initial C–C distances (Table S1), with Cu–IL (SH) exhibiting the shortest separation between reactive carbon atoms. These optimized structural features collectively lower the reaction barrier and stabilize the transition state, thereby facilitating C–C bond formation. At the final state, the C–C bond in the Cu–IL (SH) system remains the shortest (1.919 Å), reflecting the most stable coupling configuration among the catalysts examined.

In summary, IL functionalization modulates the Cu surface electronic structure through synergistic charge transfer, d-band center modulation, and orbital hybridization. These changes manifest in stronger Cu–intermediate interactions, more compact adsorption geometries, and enhanced *CO–*CO coupling, particularly in the –SH- and –COOH-functionalized systems. This mechanistic understanding provides a robust rationale for the superior C–C coupling performance observed in Cu–IL (SH).

### 3.3. IL-Mediated Modulation of Coupling Barriers and Product Selectivity in CO_2_RR on Cu-Based Catalysts—Dual Modulation of Thermodynamics and Kinetics in C–C Coupling by ILs

To elucidate the key role of ILs in modulating the rate-determining C–C coupling step during CO_2_ electroreduction on Cu-based catalysts, we performed comprehensive thermodynamic and kinetic calculations on pristine Cu and three IL-modified Cu surfaces. The transition state configurations and charge redistributions of the intermediates were obtained for the four catalyst systems, revealing the dynamic evolution of charge density on *CO intermediates throughout the reaction trajectory—from the initial state to transition state and final coupled state.

**Figure 6:**
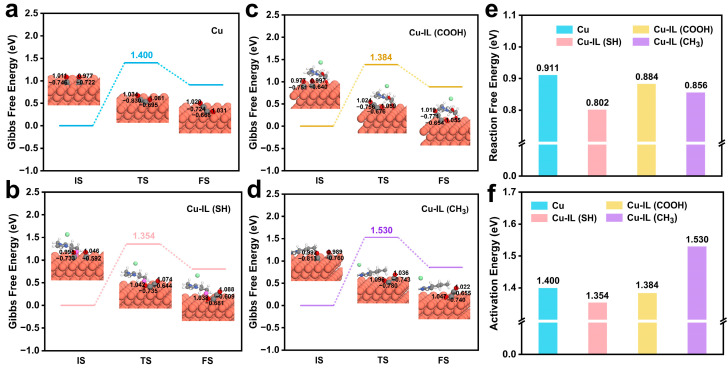
(**a**–**d**) Gibbs free energy of *CO + *CO → *COCO. (**a**) Cu, (**b**) Cu–IL (SH), (**c**) Cu–IL (COOH), and (**d**) Cu–IL (CH_3_). (**e**) Reaction free energy of *CO + *CO → *COCO. (**f**) Activation energy of *CO + *CO → *COCO.

From a thermodynamic perspective, the reaction ΔG diagram for *CO–*CO coupling on pristine Cu was calculated to be 0.911 eV (Figure 6e). Upon IL modification, this value significantly decreased to 0.884 eV for Cu–IL (COOH) and 0.856 eV for Cu–IL (CH_3_), respectively, with Cu–IL (SH) achieving the most favorable thermodynamics energy of 0.802 eV. This trend suggests that IL-induced interfacial environments can lower the thermodynamic barrier for *CO–*CO coupling, with Cu–IL (SH) exhibiting the most pronounced enhancement.

Particularly, to probe the kinetic effects of ILs, NEB was employed to accurately map the minimum energy pathways (MEPs) and obtain the corresponding activation energy barriers for pristine Cu and IL-modified systems. As shown in Figure 6a–d,f and Figure S9, among all systems, Cu–IL (SH) exhibits the lowest activation energy barrier of 1.354 eV, indicating the most favorable kinetics for *CO–*CO coupling. In stark contrast, Cu–IL (CH_3_) shows the highest barrier of 1.530 eV, reflecting a kinetically unfavorable pathway. Cu–IL (COOH) exhibits a moderately reduced barrier of 1.384 eV, falling between the values of pristine Cu and the most active Cu–IL (SH) system. These results demonstrate that ILs, particularly those bearing polar or coordinating groups, can simultaneously reduce both the reaction energy and the activation barrier, thereby enhancing both the thermodynamic driving force and the kinetic feasibility of C–C bond formation.

Structural analysis further supports these findings (Table S1). Among all systems, Cu–IL (SH) exhibits the shortest C–C bond length at the transition state (1.675 Å), reflecting a stronger and more stable coupling interaction between the two *CO intermediates. This structural feature correlates well with the enhanced thermodynamic driving force combined and a lowered activation barrier, which highlights the dual role of ILs in facilitating C–C bond formation.

Notably, the charge asymmetry between the two *CO intermediates induced by ILs at the adsorption sites strongly correlates with the efficiency of C–C coupling. In the Cu–IL (SH) system, the two *CO moieties exhibit the most uneven charge difference (0.192 e), compared to a smaller difference of 0.131 e in Cu–IL (COOH) and a nearly symmetrical charge distribution in the pristine Cu system (Table S2). This charge imbalance likely breaks the repulsive symmetry between two negatively charged *CO species, promoting directional electron flow, orbital overlap, and dipole-assisted coupling.

Taken together, these findings highlight a dual promotional effect of ILs on C–C coupling, as follows: (i) through the thermodynamic stabilization of the final *COCO product, and (ii) through kinetic acceleration by lowering the transition-state energy barrier. Among the systems studied, Cu–IL (SH) exhibits the most advantageous combination of charge asymmetry, transition-state geometry, and energy profile, making it the most promising candidate for selective CO_2_-to-C_2_ conversion.

### 3.4. IL-Guided Pathway Divergence and Suppression of Hydrogen Evolution Reaction of Cu-Based Catalysts for CO_2_RR Products

#### 3.4.1. Competitive Reaction Pathways Toward C_1_ and C_2_ Products of CO_2_RR on IL-Modified Cu

Building on their role in enhancing *CO–*CO coupling, ILs also regulate the SDS of product formation in CO_2_RR. To further reveal the intrinsic role of ILs in regulating product selectivity, we conducted a comparative calculation of the C_2_ (C_2_H_4_ and C_2_H_5_OH) and C_1_ product (CH_3_OH) formation pathways, as well as the competing HER on pristine and IL-modified Cu surfaces. After the formation of the *CO intermediate, two distinct and competitive pathways become accessible; hydrogenation generates *CHO species that drive C_1_ product formation such as methane, while coupling reactions produce *COCO—the crucial precursor for C_2_ compound synthesis. This branching point constitutes the SDS of CO_2_RR. The relative Gibbs free energies of these two competing pathways govern the product distribution.

**Figure 7:**
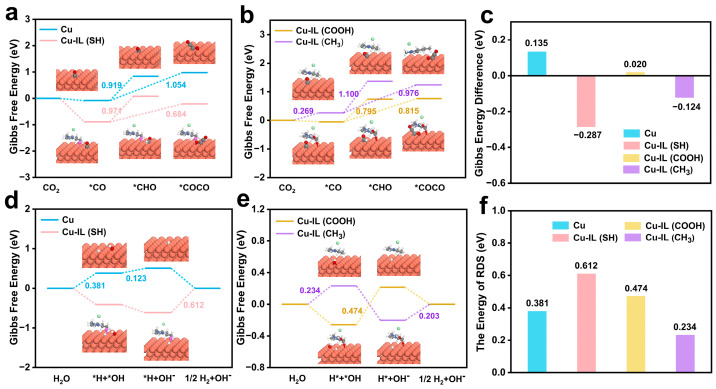
(**a**,**b**) Gibbs free energy of *CO → *CHO and *CO → *COCO on Cu, Cu–IL (SH), Cu-IL (COOH), and Cu-IL (CH_3_). (**c**) The difference of Gibbs free energy between the *CO–*CO coupling and *CO hydrogenation steps. (**d**,**e**) Gibbs free energy of HER on Cu, Cu–IL (SH), Cu-IL (COOH), and Cu-IL (CH_3_). (**f**) The energy of RDS of HER.

As shown in Figure 7a,b and Figure S10, the free energy change for *CO → *CHO on pristine Cu is 0.919 eV, which increases to 0.971 eV and 1.100 eV for Cu–IL (SH) and Cu–IL (CH_3_), respectively, indicating a thermodynamic disfavoring of C_1_ formation. In contrast, Cu–IL (COOH) lowers the energy of this step to 0.795 eV, thereby promoting C_1_ product formation. Meanwhile, for the *CO–*CO coupling step, all IL-modified systems exhibit reduced reaction free energies compared to pristine Cu. Among them, Cu–IL (SH) exhibits the most favorable thermodynamic bias toward C_2_ product formation, with a Gibbs energy difference (∆G) of −0.287 eV between the *CO–*CO coupling and *CO hydrogenation steps. The corresponding ∆G values for Cu–IL (CH_3_), Cu–IL (COOH), and pristine Cu are −0.124 eV, 0.020 eV, and 0.135 eV, respectively (Figure 7c).

#### 3.4.2. Suppression of HER

In addition to regulating the selectivity-determining step between the C_1_ and C_2_ pathways, ILs also play a pivotal role in suppressing the HER, a major side reaction that competes with CO_2_ reduction. HER not only consumes electrons and protons that would otherwise contribute to CO_2_ reduction, but also perturbs the interfacial microenvironment, altering the pH, surface potential, and charge density, all of which can influence the stability and reactivity of CO_2_RR intermediates. Consequently, uncontrolled HER lowers the C_2_H_4_ and C_2_H_5_OH yield, complicates product separation, and compromises energy efficiency. It is equally critical to evaluate their influence on HER, which can profoundly impact product selectivity and faradaic efficiency. We evaluated the HER pathways by calculating the Gibbs free energy profiles for pristine and IL-modified Cu surfaces (Figure 7d,e). The reaction pathway is divided into three thermodynamically distinct steps: (i) water dissociation (*H_2_O → *H + *OH); (ii) *OH desorption (*OH → OH^−^); and (iii) hydrogen evolution. The initial water dissociation steps are thermodynamically facilitated on all IL-functionalized surfaces compared to that on pristine Cu, including Cu–IL (SH), Cu–IL (COOH), and Cu–IL (CH_3_), indicating that ILs’ modification promotes the surface availability of reactive hydrogen species. However, the subsequent desorption steps exhibit significant thermodynamic variation.

The RDS of HER significantly depends on the surface functionalization of Cu with different ILs (Figure 7f). On pristine Cu, the RDS is the initial water dissociation step, with a Gibbs free energy change of 0.381 eV, indicating moderate HER activity in the absence of interfacial tuning. Upon ILs modification, distinct shifts in the RDS are observed. Cu–IL (SH) presents the highest Gibbs free energy change of 0.612 eV among all systems, associated with the hydrogen evolution step involving the recombination and release of surface-bound hydrogen. As the thermodynamic rate-limiting step in the HER pathway, it reflects the pronounced suppression of HER on the Cu–IL (SH) surface. In contrast, the Cu–IL (COOH) system exhibits its highest Gibbs free energy change (0.474 eV) during hydroxide desorption, indicating that the removal of surface-bound *OH constitutes the thermodynamic rate-limiting step. This suggests that HER is suppressed by the stabilization of *OH intermediates, which delays site regeneration for subsequent proton transfer. In contrast, all elementary steps in the Cu–IL (CH_3_) system along the HER pathway exhibit low Gibbs free energy changes, with the highest being only 0.234 eV. The uniformly low energy barriers suggest a facile HER process without a clear thermodynamic bottleneck.

These findings underscore the critical role of IL functional groups in modulating the thermodynamic bottleneck of the HER pathway. Polar moieties such as –SH and –COOH elevate the rate-limiting free energy by stabilizing adsorbed hydrogen or hydroxide species, thereby suppressing HER through distinct desorption-limited mechanisms. In contrast, the nonpolar –CH_3_ group exerts minimal impact on the HER energy landscape, leaving the pathway largely unimpeded. The ability of –SH- and –COOH-functionalized ILs to increase the energetic threshold of the rate-determining step provides a mechanistic basis for their enhanced CO_2_RR selectivity, achieved through the effective suppression of the competing HER process.

## 4. Conclusions

This work systematically elucidates the mechanism of 1-(3-X-propyl)-3-methylimidazolium-based ILs in modulating the electrocatalytic behavior of Cu electrodes toward C_2_H_4_ and C_2_H_5_OH formation in CO_2_RR. Theoretical investigations reveal that ILs bearing different terminal functional groups can trigger the interfacial electronic reconstruction, change the position of the d-band center, and affect the adsorption strength and configuration of key intermediates. In particular, Cu–IL (SH) exhibits the most favorable electronic modulation, enhancing *CO adsorption, stabilizing the *CO–*CO coupling configuration through stronger Cu–C interactions, and ultimately promoting efficient C–C bond formation. Thermodynamic and kinetic analyses further confirm that Cu–IL (SH) has the lowest reaction free energy and activation barrier for the rate-determining C–C coupling step, underscoring the pivotal role of electronic structure regulation in enhancing catalytic performance. In addition, IL modification also substantially improves the product selectivity by directing the reaction pathway towards C_2_H_4_ and C_2_H_5_OH while simultaneously suppressing undesired C_1_ formation and the competitive HER.

These findings demonstrate that the catalytic enhancement in CO_2_RR arises from a synergistic mechanism involving IL-induced interfacial electronic modulation, thermodynamic stabilization, and the kinetic promotion of the rate-determining C–C coupling step. The consistent trends observed across d-band shifts, adsorption energies, and energy barriers establish a robust structure–function relationship governed by ILs’ polarity. This study not only advances the fundamental understanding of Cu–IL catalyst interactions but also provides a broadly applicable framework for the rational design of IL-modified electrocatalysts toward highly selective and energy-efficient CO_2_ conversion. While this work focuses on Cu as a model system, we envision that the rational design of ionic liquid functional groups—considering the dipole moment, donor/acceptor character, and binding affinity—could be extended to tailor interfacial interactions on other metallic surfaces. Such molecular-level tuning may enable the application of ionic liquid-assisted catalysis in a broader range of fields.

## Figures and Tables

**Table 1:** The bond length of Cu–C or distance of Cu to C.

	State	IS	TS	FS
Sample				
Cu		1.999 Å	1.972 Å	2.025 Å
Cu–IL (SH)		1.975 Å	1.967 Å	2.021 Å
Cu–IL (COOH)		1.990 Å	1.975 Å	2.026 Å
Cu–IL (CH_3_)		2.016 Å	1.979 Å	2.029 Å

## Data Availability

Data are contained within the article and Supplementary Materials.

## Supplementary Materials

The following supporting information can be downloaded at: https://www.mdpi.com/article/10.3390/molecules30112352/s1, Figure S1: The configuration of (a) pristine Cu, (b) Cu–IL (SH), (c) Cu–IL (COOH), and (d) Cu–IL (CH_3_) (top view); Figure S2: The configuration of (a) pristine Cu, (b) Cu–IL (SH), (c) Cu–IL (COOH), and (d) Cu–IL (CH_3_) (side view); Figure S3: Bader charge at some Cu points (a) pristine Cu, (b) Cu–IL (SH), (c) Cu–IL (COOH), and (d) Cu–IL (CH_3_); Figure S4: The configuration of initial state of C–C coupling on (a) pristine Cu, (b) Cu–IL (SH), (c) Cu–IL (COOH), and (d) Cu–IL (CH_3_) (top view); Figure S5: The configuration of initial state of C–C coupling on (a) pristine Cu, (b) Cu–IL (SH), (c) Cu–IL (COOH), and (d) Cu–IL (CH_3_) (side view); Figure S6: The configuration of final state of C–C coupling on (a) pristine Cu, (b) Cu–IL (SH), (c) Cu–IL (COOH), and (d) Cu–IL (CH_3_) (top view); Figure S7: The configuration of final state of C–C coupling on (a) pristine Cu, (b) Cu–IL (SH), (c) Cu–IL (COOH), and (d) Cu–IL (CH_3_) (side view); Figure S8: The different charges of the *CO + *CO and Cu/Cu–IL surfaces in pure Cu and three IL-modified catalyst systems in the initial state of C–C coupling, with cyan and yellow areas representing charge depletion and charge accumulation, respectively. (a) pristine Cu, (b) Cu–IL (SH), (c) Cu–IL (COOH), (d) Cu–IL (CH_3_); Figure S9: The configuration of transition state of C–C coupling on (a) pristine Cu, (b) Cu–IL (SH), (c) Cu–IL (COOH), and (d) Cu–IL (CH_3_) (side view); Figure S10: The energy of RDS of *CO–*CO coupling and *CO hydrogenation steps; Table S1: The bond length of C–C or distance of C to C; Table S2: The difference between the bader charges of two *CO.
