# Supplementary material for: Electronic Modulation of Cu Catalytic Interfaces by Functionalized Ionic Liquids for Enhanced CO2 Reduction
Source: Molecules. 2025 May 28;30(11):2352. doi: 10.3390/molecules30112352 (PMC12155725; doi:10.3390/molecules30112352)
Supplement: Supplementary file 1 [file molecules-30-02352-s001.zip › molecules-3632101-supplementary.pdf]

Electronic Modulation of Cu Catalytic Interfaces by Functionalized Ionic Liquids for Enhanced CO_2 Reduction

Chuanhui Wang [1], Wei Zhou [1], Jiamin Ma [1], Zhi Wang [2,*], Congyun Zhang [1,*]

[1] School of Environment and Geography, Qingdao University, Qingdao 266071, China; wch2022020768@163.com (C.W.); 13770594749@163.com (W.Z.); majiamin07@163.com (J.M.).

[2] School of Materials Science and Engineering, North University of China, Taiyuan 030051, People's Republic of China.

*** Correspondence:** shikouri@163.com (Z.W.); zhangcy@qdu.edu.cn (C.Z.).

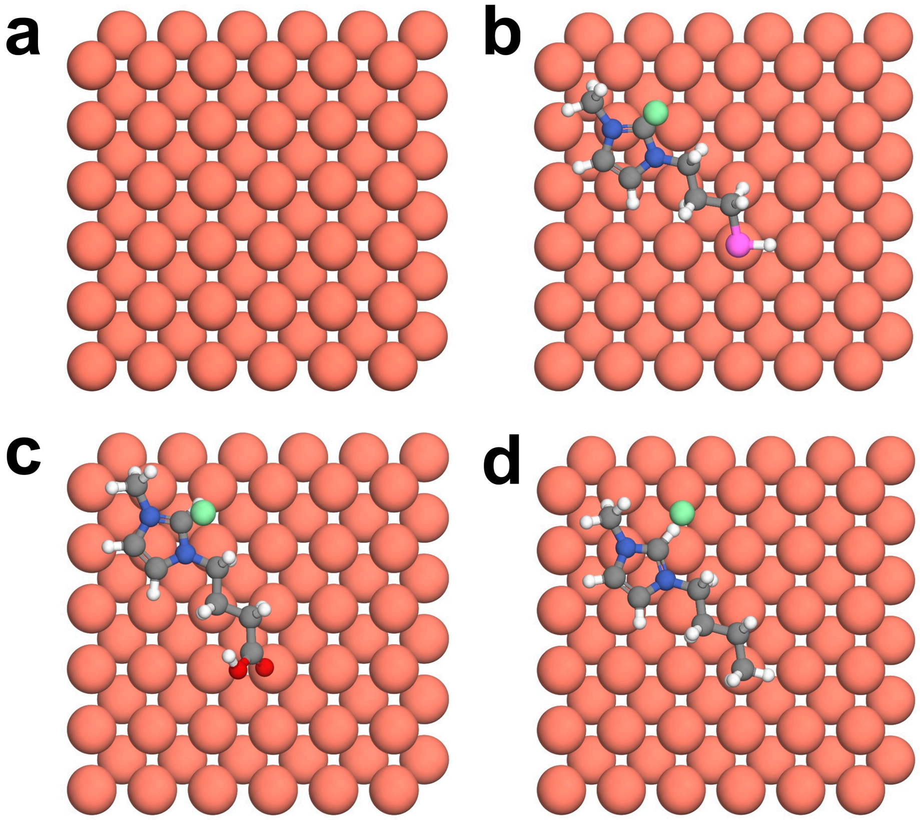

Figure S1. The configuration of (a) pristine Cu, (b) Cu–IL (SH), (c) Cu–IL (COOH) and (d) Cu–IL (CH_3) (top view).

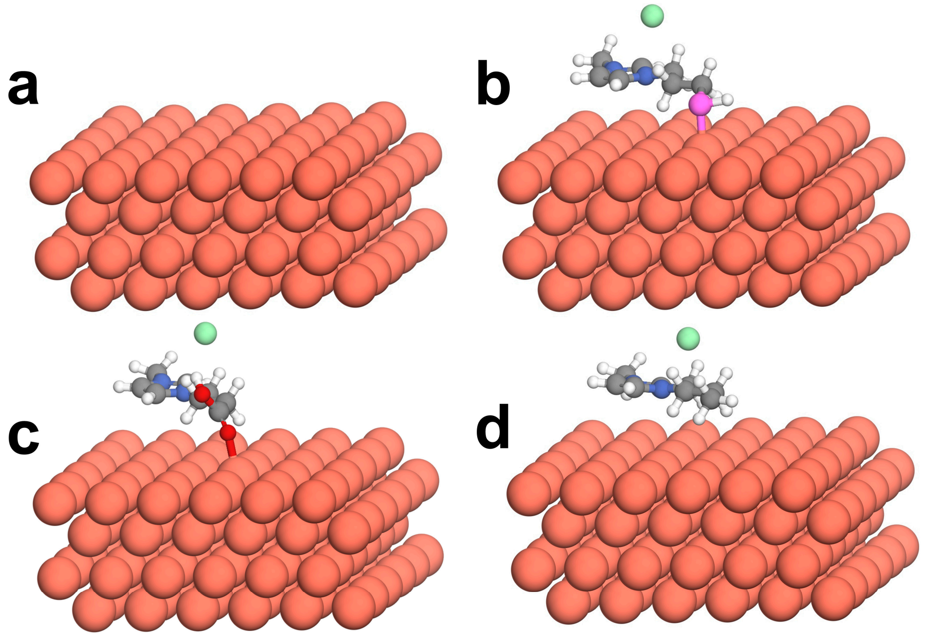

Figure S2. The configuration of (a) pristine Cu, (b) Cu–IL (SH), (c) Cu–IL (COOH) and (d) Cu–IL (CH_3) (side view).

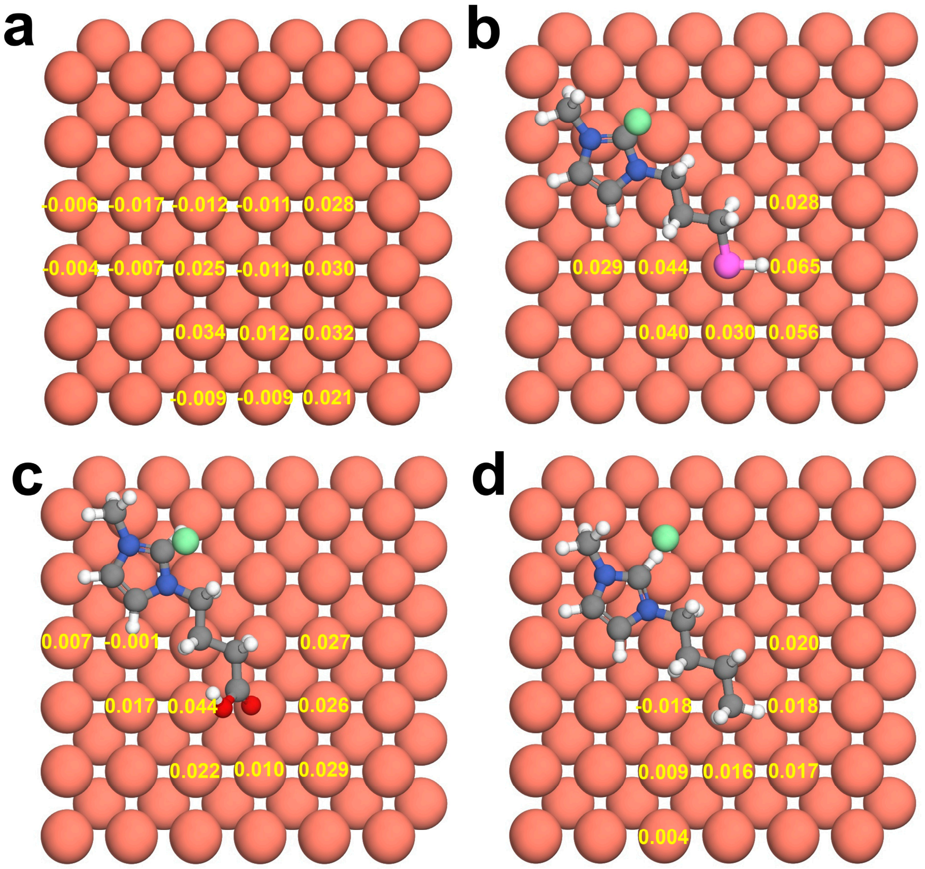

Figure S3. Bader charge at some Cu points (a) pristine Cu, (b) Cu–IL (SH), (c) Cu–IL (COOH) and (d) Cu–IL (CH_3).

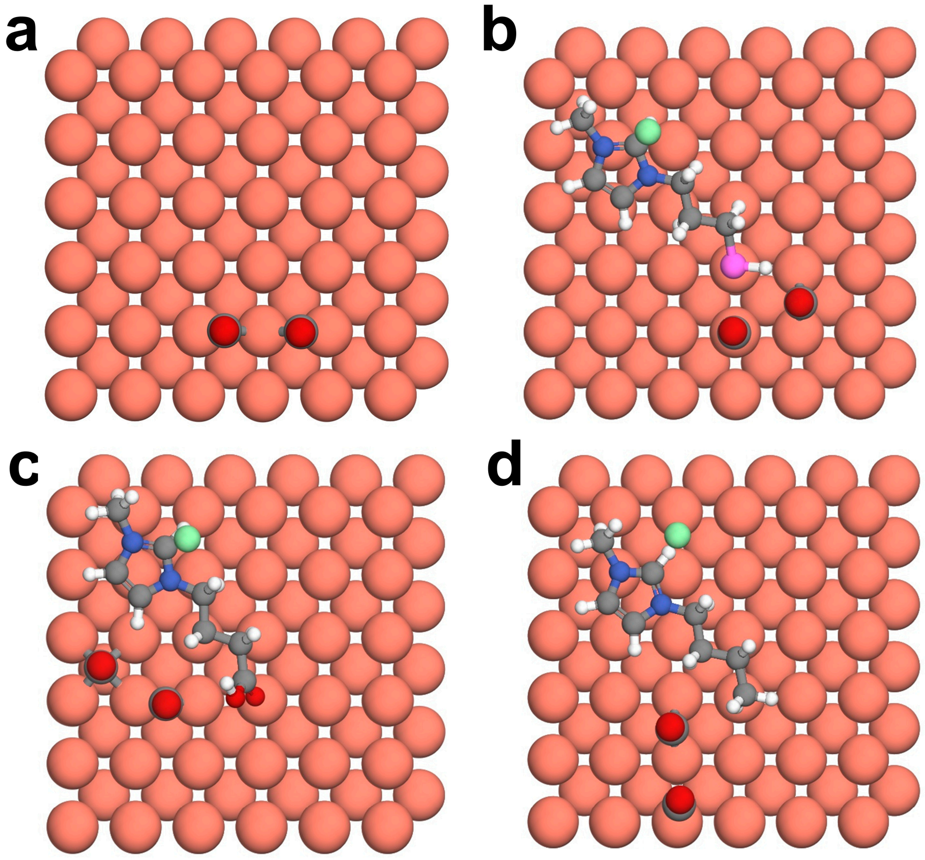

Figure S4. The configuration of initial state of C–C coupling on (a) pristine Cu, (b) Cu–IL (SH), (c) Cu–IL (COOH) and (d) Cu–IL (CH_3) (top view).

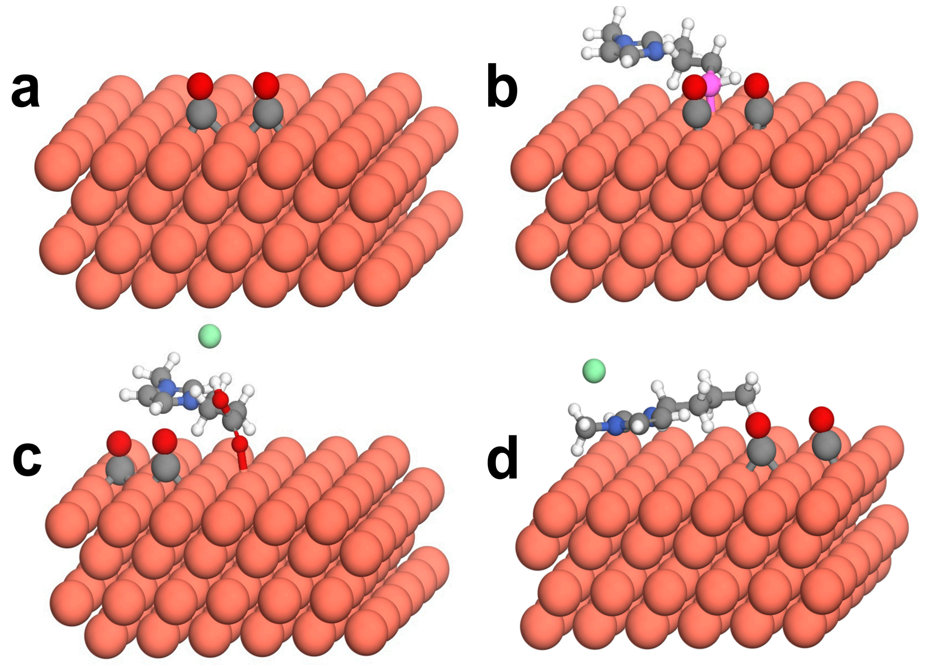

Figure S5. The configuration of initial state of C–C coupling on (a) pristine Cu, (b) Cu–IL (SH), (c) Cu–IL (COOH) and (d) Cu–IL (CH_3) (side view).

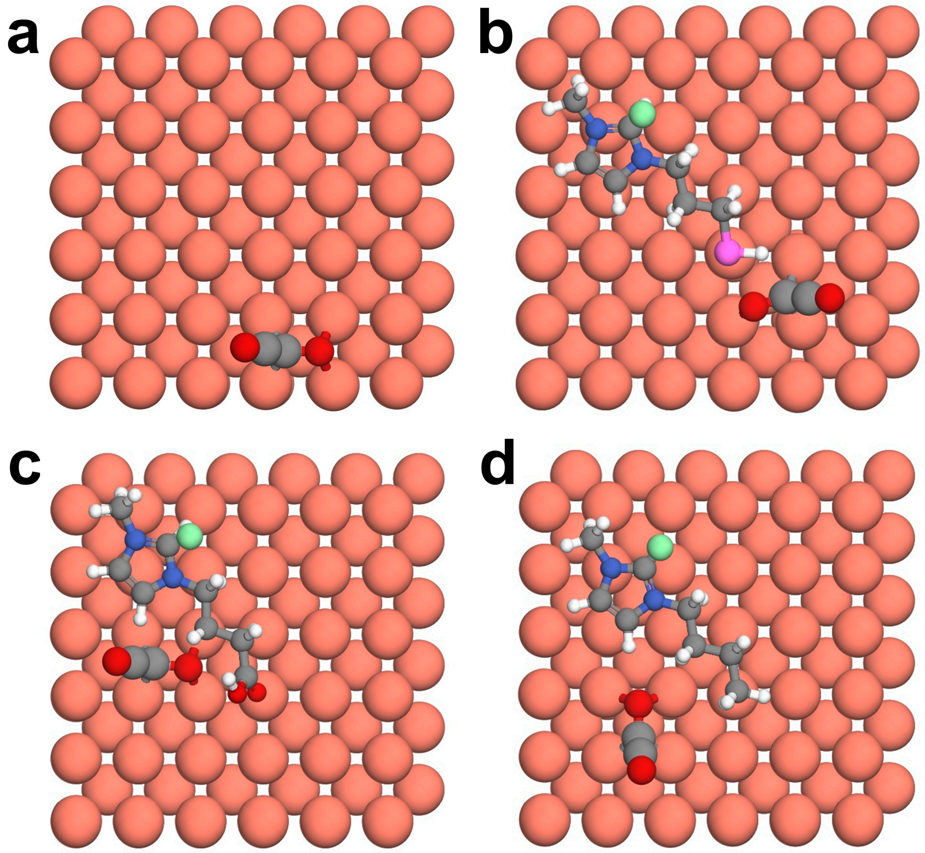

Figure S6. The configuration of final state of C–C coupling on (a) pristine Cu, (b) Cu–IL (SH), (c) Cu–IL (COOH) and (d) Cu–IL (CH_3) (top view).

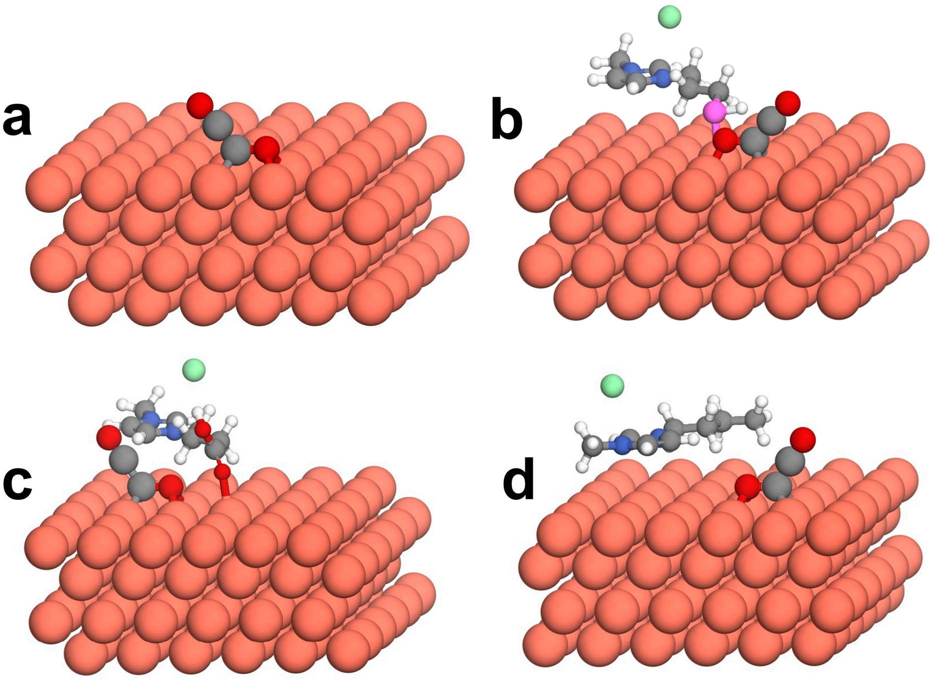

Figure S7. The configuration of final state of C–C coupling on (a) pristine Cu, (b) Cu–IL (SH), (c) Cu–IL (COOH) and (d) Cu–IL (CH_3) (side view).

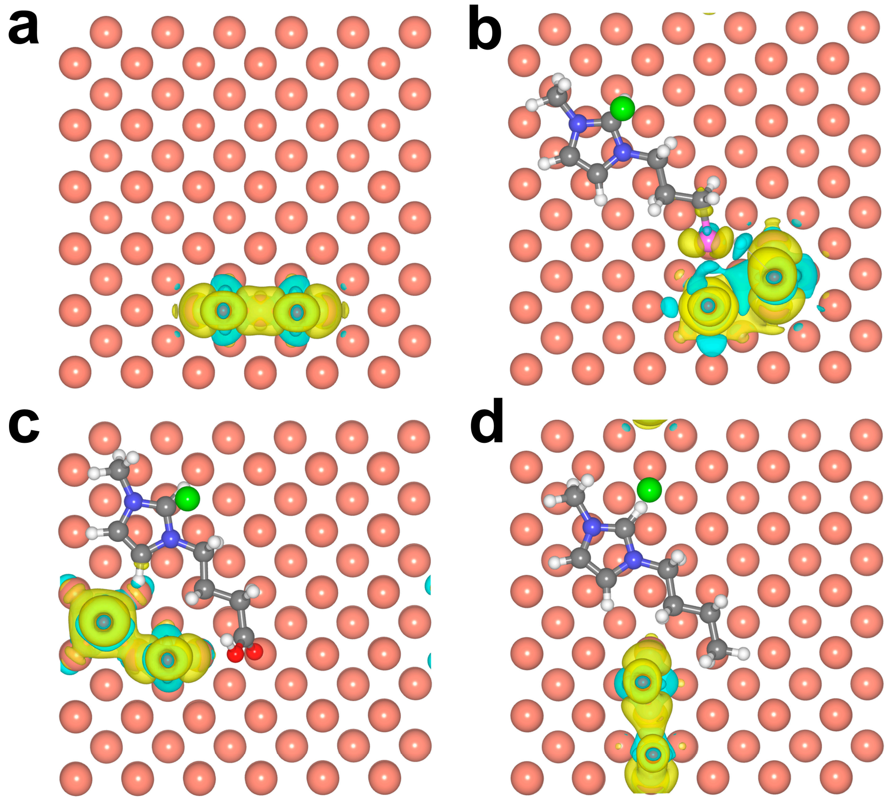

Figure S8. The difference charges of the *CO + *CO and Cu/ Cu–IL surfaces in pure Cu and three ILs–modified catalyst systems in the initial state of C–C coupling, with cyan and yellow areas representing charge depletion and charge accumulation, respectively. (a) pristine Cu, (b) Cu–IL (SH), (c) Cu–IL (COOH), (d) Cu–IL (CH_3).

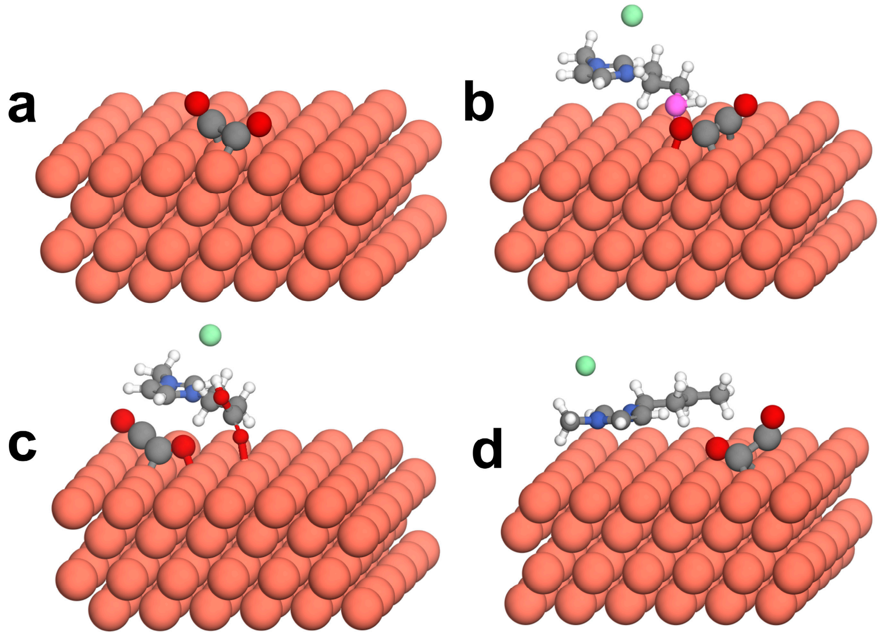

Figure S9. The configuration of transition state of C–C coupling on (a) pristine Cu, (b) Cu–IL (SH), (c) Cu–IL (COOH) and (d) Cu–IL (CH_3) (side view).

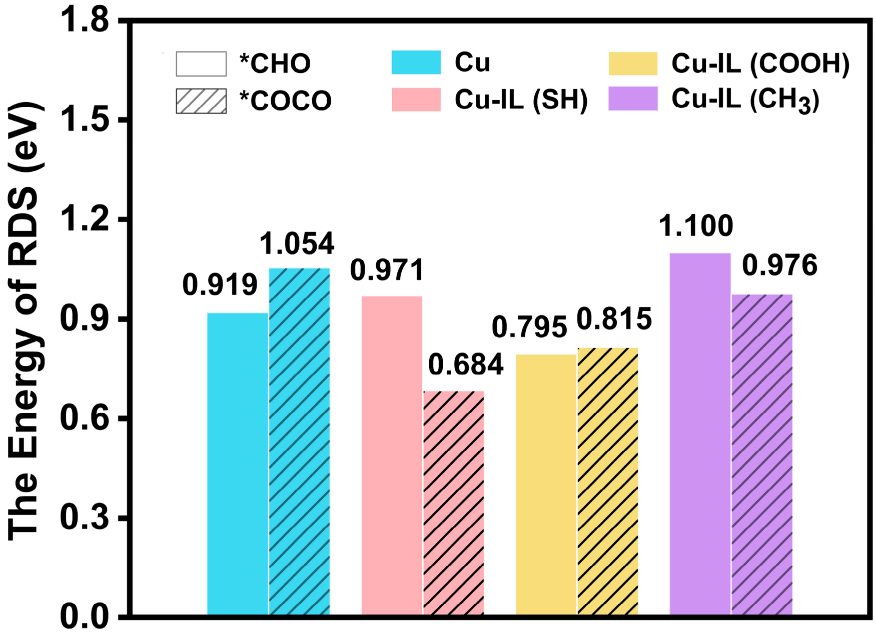

Figure S10. The energy of RDS of *CO – *CO coupling and *CO hydrogenation steps.

Table S1. The bond length of C–C or distance of C to C.

State / sample	IS	TS	FS
Cu	3.065 Å	1.759 Å	1.342 Å
Cu–IL (SH)	3.013 Å	1.675 Å	1.346 Å
Cu–IL (COOH)	3.058 Å	1.697 Å	1.419 Å
Cu–IL (CH_3)	3.135 Å	1.770 Å	1.361 Å

Table S2. The difference between the bader charges of two *CO.

sample	The difference of bader charge
Cu	0.009 e
Cu–IL (SH)	0.192 e
Cu–IL(COOH)	0.131 e
Cu–IL (CH_3)	0.051 e
